# Hydrolytic Degradation of Porous Crosslinked Poly(ε-Caprolactone) Synthesized by High Internal Phase Emulsion Templating

**DOI:** 10.3390/polym12081849

**Published:** 2020-08-18

**Authors:** Nejla Benyahia Erdal, Gabriela Albara Lando, Anilkumar Yadav, Rajiv K. Srivastava, Minna Hakkarainen

**Affiliations:** 1 Department of Fibre and Polymer Technology, KTH Royal Institute of Technology, 100 44 Stockholm, Sweden; nejla@kth.se (N.B.E.); gabrial@kth.se (G.A.L.); 2 Department of Physical Chemistry, Institute of Chemistry, Universidade Federal de Rio Grande do Sul (UFRGS), Av. Bento Gonçalves, 9500, Porto Alegre CEP 91501-970, RS, Brazil; 3 Department of Textile and Fibre Engineering, Indian Institute of Technology Delhi, Hauz Khas, New Delhi 110016, India; anil20yadav@gmail.com (A.Y.); rajiv@textile.iitd.ac.in (R.K.S.)

**Keywords:** polycaprolactone, high internal polymerization emulsion, hydrolytic degradation, scaffold

## Abstract

Porous poly(ε-caprolactone) (PCL) scaffolds were fabricated using the high internal polymerization emulsion (HIPE) technique. Bis(ε-caprolactone-4-yl) (BCY) was utilized as crosslinker. The crosslinking density and the volume fraction of the dispersed phase were varied in order to study the potential effect of these parameters on the hydrolytic degradation at 37 °C and 60 °C. After different hydrolysis times the remaining solid samples were analyzed by Fourier transform infrared spectroscopy (FTIR) and scanning electron microscopy (SEM), while the degradation products in the aqueous aging solutions were analyzed by laser desorption ionization-mass spectrometry (LDI-MS). The effect of temperature on the degradation process and release of degradation products was, as expected, significant. The temperature effect was also shown by FTIR analysis that displayed a pronounced increase in the intensity of the hydroxyl-group absorption band after 70 days of hydrolysis at 60 °C indicating significant cleavage of the polymer chains. LDI-MS analysis proved the release of oligomers ranging from dimers to hexamers. The product patterns were similar, but the relative *m*/*z* signal intensities increased with increasing time, temperature and crosslinking density, indicating larger amounts of released products. The latter is probably due to the decreasing degree of crystallinity as a function of amount of crosslinker. The porous structure and morphology of the scaffolds were lost during the aging. The higher the crosslinking density, the longer the scaffolds retained their original porous structure and morphology.

## 1. Introduction

Biocompatible, bioresorbable and degradable polymers have become important attributes for polymer applications during the last two decades, especially in the field of tissue engineering [1]. Aliphatic polyesters have been studied extensively as bioresorbable three-dimensional (3D) scaffolds, drug delivery vehicles, films and fibers due to their biodegradable and biocompatible character [2]. Among aliphatic polyesters, poly(ε-caprolactone) (PCL) is a semicrystalline polyester synthesized by ring-opening polymerization of the cyclic lactone in the presence of a catalyst [3]. With a glass transition temperature of −60 °C and a melting temperature of 56–65 °C, PCL has shown to possess superior rheological properties making it easily fabricated and tailored by different methods into an array of scaffolds [1,4,5,6]. One essential issue for biodegradable polymers in the tissue engineering field is controlled degradation rate in combination with nature and amount of released degradation products [7,8]. Degradation of aliphatic polyesters in vitro and in vivo involves the random hydrolytic scission of ester bonds rendering the release of water-soluble oligomers and monomeric hydroxyl acids [9,10]. The hydrolytic degradation of PCL is, however, considerably slower compared to other aliphatic polyesters due to its hydrophobicity and high crystallinity [11].

The abiotic and biotic degradation processes of aliphatic polyesters have been investigated thoroughly and the possibility of tuning the degradation rate and product pattern by the macromolecular design has also been reported [12,13]. Copolymerization and crosslinking can enhance the susceptibility to degradation by increasing the hydrophilicity and by decreasing the crystallinity [14,15]. As an example, ε-caprolactone (CL) and 1,5 dioxepan-2-one (DXO) were polymerized into different macromolecular structures, i.e., DXO/CL/DXO triblocks, CL/DXO multiblocks, and random crosslinked CL/DXO copolymers. It was shown that the triblock copolymer yielded the largest amount of both monomeric hydrolysis products and water-soluble oligomers due to the long hydrophilic DXO block, while the crosslinking resulted in release of 6-hydroxyhexanoic acid and caused a shift from water-soluble oligomers towards the monomeric hydroxyacids [13]. In a more recent study, bis-(ε-caprolactone-4-yl) (BCY) was used to crosslink PCL fibers in situ during melt spinning [16]. BCY was incorporated to enhance the strength of the fibers and to prevent molecular weight reduction during melt spinning. The effects of crosslinking, crosslinking density and processing conditions on the hydrolytic degradation of the PCL fibers were studied and it was demonstrated that the presence of BCY had a profound effect on the degradation together with the processing conditions [17].

The internal microarchitecture of the scaffold is another key element in tissue engineering where highly interconnected pores are desired. Currently there are several methods to prepare porous, biodegradable 3D scaffolds including different additive manufacturing techniques, such as fused deposition modeling and stereolithography (SLA), and other methods like electrospinning, solvent casting, particulate leaching and thermally induced phase separation (TIPS) [18]. Another increasingly exploited method is high internal phase emulsion (HIPE) that typically involves emulsions containing a dispersed phase above 74% of the total emulsion volume [5,19,20]. The polymerization of the continuous (non-droplet) phase with monomers and the removal of the internal phase creates highly porous materials known as polyHIPEs [21]. Porous polyHIPE foams containing PCL have been prepared through water-in-oil (w/o) HIPE using free radical polymerization of a PCL macromonomer either alone or together with a comonomer (styrene or methyl methacrylate) [22]. While the internal phase of w/o HIPE is commonly comprised of water, the continuous phase can consist of different monomer(s), surfactant(s), initiators and emulsifiers.

It is of interest to understand if and to what extent the degree of crosslinking and fraction of dispersed phase influences the hydrolytic degradation of PCL-based porous scaffolds synthesized via HIPE polymerization and in-situ crosslinked with BCY. The effects of increasing volume fraction of internal phase and amount of crosslinker on hydrolytic degradation and degradation product profiles of porous PCL scaffolds were therefore monitored by laser desorption ionization-mass spectrometry (LDI-MS), Fourier transform infrared spectroscopy (FTIR) and scanning electron microscopy (SEM) during different time periods at 37 °C and 60 °C. The matrix changes and release of water-soluble products were evaluated by several techniques.

## 2. Materials and Methods

### 2.1. Materials

Water (LC-MS grade) was obtained from Merck (Sweden). ε-Caprolactone (CL, 99%) was purchased from Thermo Fisher Scientific, Mumbai, India. Pluronic F127 (–[CH_2_CH_2_O]_100_–[CH_2_CH(CH)_3_O]_70_–[CH_2_CH_2_O]_100_), Span 80 (sorbitan monooleate), stannous octoate (Sn(Oct)_2_), (92.5–100%), hexadecane and poly(ε-caprolactone) (PCL, reported M_n_ = 80,000 g/mol by supplier) were procured from Sigma Aldrich, Bengaluru, India. The crosslinking monomer, bis(ε-caprolactone-4-yl) (BCY), was synthesized by previously reported procedure from precursor, 4,4′-biscyclohexanone (>98%) supplied by TCI Chemicals Pvt. Ltd., Chennai, India [16]. All chemicals were used as received.

### 2.2. Preparation of PCL-Based PolyHIPEs

A non-aqueous HIPE was first prepared by dispersing hexadecane in continuous phase comprised of monomer (CL), crosslinker (BCY), emulsifier (F127, accounting for 60 wt% of monomers) and catalyst (Sn(Oct)_2_). The resulting HIPE was polymerized at 120 °C for 8 h in an oil bath. The porous scaffolds thus obtained were Soxhlet extracted with n-hexane and methanol followed by drying under vacuum at 40 °C. Scaffolds were stored under vacuum at room temperature for further characterization. Several scaffolds with different volume fractions of dispersed phase (*ϕ_2_)* and theoretical crosslink densities (*D_x_*) were prepared by changing the amount of hexadecane or BCY in the emulsion, see Table 1. The porosity of the scaffolds was determined in the previous study and varied from 42 to 74% [23]. Higher *ϕ*_2_ increased the porosity, while the porosity was slightly decreased with increasing *D_x_*.

A porous non-crosslinked scaffold of commercial PCL was prepared to be used as a reference. A 10 wt% solution of PCL was prepared in toluene and span 80 (20 wt%) was later dissolved in it. A solution of calcium chloride (1 wt%) in deionized water (Millipore, Bengaluru, India) was added dropwise to the oil phase under continuous stirring. The volume fraction of continuous to internal phase was maintained at 0.26/0.74. The obtained HIPE was lyophilized followed by thorough washing with deionized water and methanol. The scaffold was finally dried under vacuum at room temperature and stored for further analysis. The neat PCL polyHIPE scaffold was designated as PCL-01.

The density (*ρ*_2_) of PCL scaffolds was determined from the mass-to-volume relation using the samples of identical size. The porosity of samples was calculated from Equation (1), considering the density of pure PCL (density, *ρ*_1_ = 1.145 g/cm^3^).

(1) $$Porosity\;(\%)\;=\;\frac{\left(\rho_{1}-\rho_{2}\right)}{\rho_{1}}\times 100$$

The values of density and porosity of crosslinked PCL scaffolds are summarized in Table 2. The values of density and porosity for sample PCL-01 were not determined as it was difficult to obtain samples of identical size and shape for measurements. However, based on the dispersed phase content, the porosity should be 74%.

### 2.3. Differential Scanning Calorimetry (DSC)

Thermal characteristics of PCL scaffolds were studied through differential scanning calorimetry (DSC, Q2000, TA Instruments, New Castle, DE, USA). Samples were subjected to distinct thermal scans at a rate of 10 °C/min. Known quantity of sample was first heated from 30 to 100 °C, then cooled down to −60 °C and finally reheated up to 100 °C. PCL (*M_n_* = 80,000 g/mol) purchased form Sigma Aldrich (Begaluru, India) was utilized as reference. The temperature of melting (*T_m_*) and respective change in heat of fusion (Δ*H_f_*) were determined from second heating cycle. The relative crystallinity of PCL scaffolds was calculated using Equation (2), where $\Delta H_{f}^{0}$ of 136 J/g corresponds to heat of fusion of 100% crystalline PCL sample [24,25].

(2) $$\mathrm{Crystallinity}\;(\%)\;=\;\frac{\Delta H_{f}}{\Delta H_{f}^{0}}\times \;100$$

### 2.4. Hydrolytic Degradation

Approximately 10 mg of PCL scaffold and 2 mL of LC-MS grade water were placed in 20-mL glass vials sealed with butyl/PTFE septa and aluminum lids. The samples used for the degradation study were cubic discs with the dimensions 3 × 3 × 2 mm^3^. The degradation study was carried out at 37 °C to simulate body temperature and at 60 °C to accelerate the slow hydrolysis process of PCL. The samples were aged in thermostatically controlled ovens for 7, 21 and 70 days. Triplicate samples were made for each crosslinked polymer sample. After degradation, the samples were withdrawn from the water medium and dried under vacuum at room temperature. Both liquid and solid phases were kept for further analysis.

### 2.5. Laser Desorption Ionization-Mass Spectrometry (LDI-MS)

The water medium after each hydrolysis time and temperature was analyzed using a Bruker UltraFlex time-of-flight (TOF) mass spectrometer (Bruker Daltonics, Bremen, Germany) with a SCOUT-MTP ion source in reflector mode, equipped with a 337 nm nitrogen laser. The analyses were carried out without matrix. The water medium was filtered with PTFE filters (13 mm × 0.45 μm) to remove any possible solid residues from the PCL degradation. An amount of 1 μL of water medium was directly spotted on the target plate three times (after letting it dry each time). The mass-to-charge (*m*/*z*) ratio range was set to 60–2000 with a reflector voltage of 26.3 kV and an accelerated voltage of 25 kV. The obtained spectrum for each sample is an accumulation of 27 spectra with 1000 laser shots at 9 different spots.

### 2.6. Fourier Transform Infrared Spectroscopy (FTIR)

FTIR spectroscopy measurements (4000 to 600 cm^−1^, 4 cm^−1^ spectral resolution, 12 scans in duplicate) were performed using Perkin Elmer Spectrum 100 with Attenuated Total Reflectance (ATR) mode.

### 2.7. Scanning Electron Microscopy (SEM)

An ultrahigh resolution field emission FE-SEM Hitachi S-4800 SEM was used to study the surface morphology of the scaffolds before and after hydrolytic degradation. The samples were sputter-coated with a gold/palladium mixture using a Cressington 208 HR Sputter Coater (Cressington Scientific Instruments, Ltd., Watford, UK) prior to the SEM observations.

## 3. Results

**Scheme 1:**
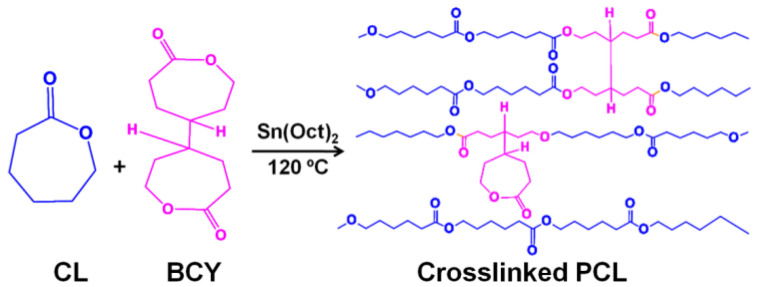
Synthesis of crosslinked PCL via single step ring opening polymerization and in-situ crosslinking.

The field of tissue engineering involves an amalgamation of cells, biomaterials and suitable bio-factors to regenerate damaged tissue and organs [26]. One of the structural components of tissue engineering is a biomaterial-based scaffold that mimics the extracellular matrix of cells. This scaffold should gradually degrade and eventually be completely resorbed as reconstructive tissue is being matured [27]. Degradation products from aliphatic polyesters may however cause an inflammatory response at the implantation site, even though they are non-toxic and excreted in the human body [28,29]. Here we investigated the degradation profile of PCL-based polyHIPE scaffolds and the influence of different amounts of BCY crosslinker and dispersed phase. Crosslinking of PCL chains with BCY was achieved via high internal phase emulsion-ring opening polymerization (HIPE-ROP) catalyzed by Sn(Oct)_2_. During the HIPE-ROP, PCL chains were covalently bound to each other through different amounts of BCY crosslinks depending on the HIPE formulations. It was earlier shown that polyHIPE of PCL scaffolds crosslinked with BCY result in promising scaffolds with significant compressive strengths and good cell viability [23]. Scheme 1 describes the polymerization of CL and in-situ crosslinking with bis-(ε-caprolactone-4-yl) (BCY) via ring opening polymerization (ROP) catalyzed using Sn(Oct)_2_ at 120 °C [16].

### 3.1. Thermal Properties and Crystallinity

**Figure 1:**
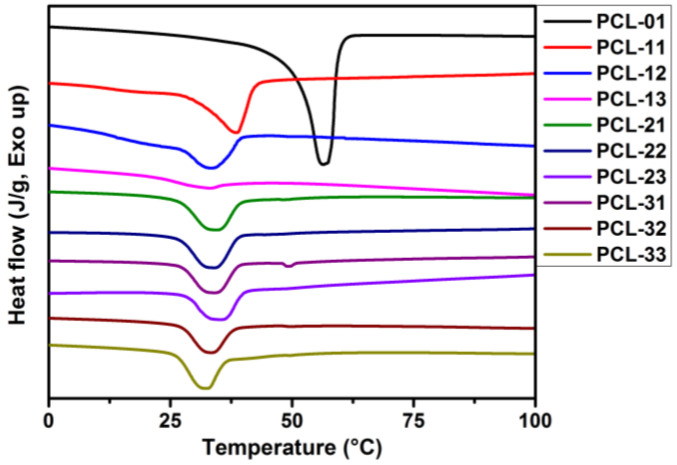
Differential scanning calorimetry (DSC) curves of the PCL scaffolds (second heating cycle).

The thermal properties, density and porosity of the scaffolds prior to hydrolytic degradation are presented in Table 2 and the DSC curves in Figure 1. The degree of crystallinity is one of the main factors controlling degradation [11], although other factors like porosity and thickness of the samples can also have considerable influence on the process [30]. Decreasing the crystallinity of a material through crosslinking makes it easier for water to penetrate the material, making the material more susceptible to degradation [13,14,15]. As seen in Table 2 there is a clear difference between the neat non-crosslinked reference PCL scaffold (PCL-01) and the crosslinked PCL scaffolds. The crystallinity of the crosslinked scaffold was significantly lower compared to the reference PCL scaffold. The differences between the different crosslinked samples were smaller, although there was a small further decrease in the degree of crystallinity as a function of increased fraction of dispersed phase during scaffold preparation (i.e., the scaffolds with higher porosity also had slightly lower crystallinity [29]). The melting temperature (*T*_m_) of all crosslinked samples was relatively similar (~32–38 °C) and clearly lower than the *T*_m_ of the non-crosslinked PCL-01 (~56 °C). As expected, the larger the amount of dispersed phase during the synthesis, the lower the density and larger the porosity of the scaffolds. Increasing crosslinking density had the opposite effect, i.e., higher crosslinking density resulted in increasing density and lower porosity, although the effect was not as pronounced as the effect of the dispersed phase.

### 3.2. Analysis of Degradation Products

**Figure 2:**
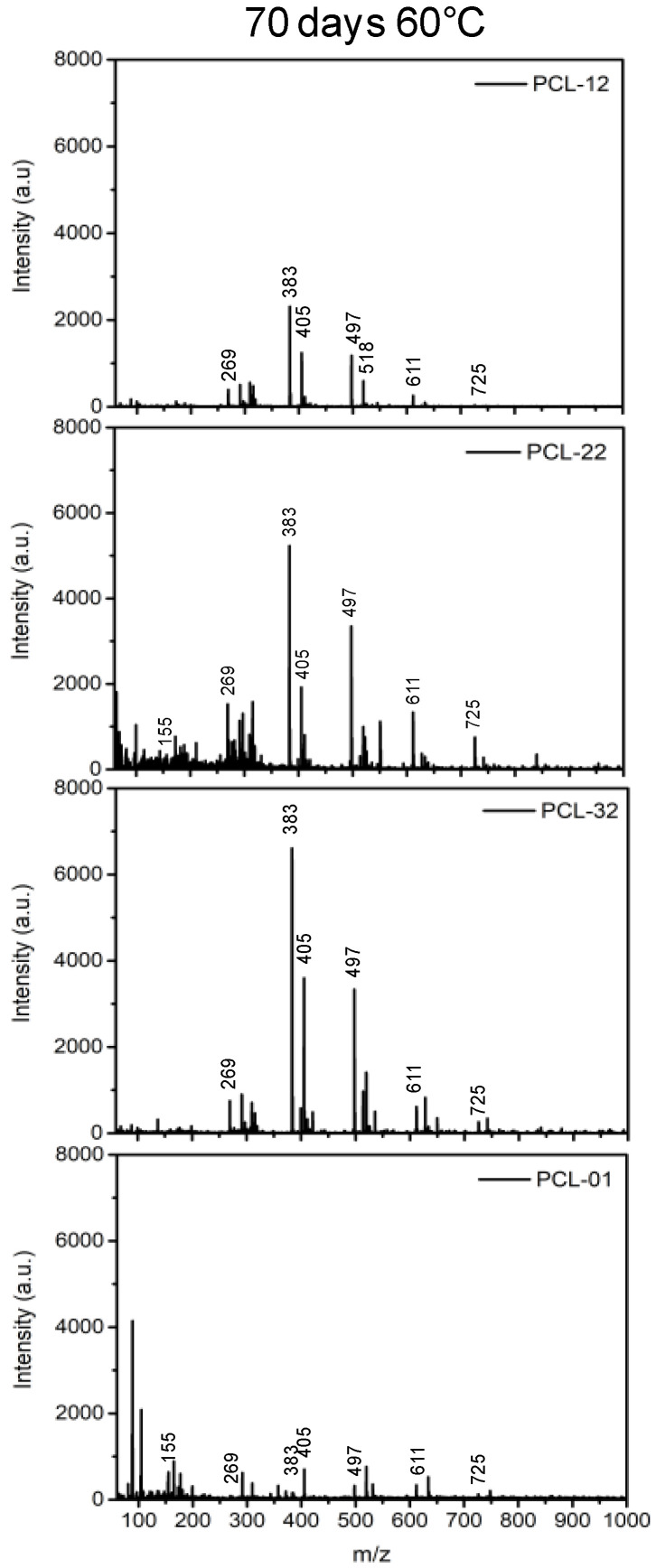
LDI-MS spectra of PCL-12, PCL-22, PCL-32 and PCL-01 after hydrolytic degradation for 70 days at 60 °C. The *m*/*z* values for the most abundant peaks are shown.

LDI-MS analysis of the water fractions after hydrolytic degradation of the PCL scaffolds crosslinked with BCY and the non-crosslinked PCL-01 scaffold all showed migration of low molecular weight compounds after one week at 60 °C. The most abundant peaks in the mass spectra corresponded to the linear 6-hydroxy acid oligomers from dimer up to hexamer. The same compounds with varying intensities were detected when the aging time was prolonged to 21 or 70 days. Figure 2 shows as an example the LDI-MS spectra of the oligomers that had migrated after hydrolytic aging of PCL-01, PCL-12, PCL-22 and PCL-32 for 70 days 60 °C. No significant differences compared to the neat scaffold PCL-01 were seen, but the peak intensities increased as a function of crosslinking density, which could be related to the decreasing degree of crystallinity. The difference in peak intensities was especially significant between the non-crosslinked PCL-01 and the crosslinked scaffolds, probably reflecting the large difference in degree of crystallinity between the non-crosslinked and crosslinked scaffolds.

Temperature had, as expected, a large impact on the degradation. None degradation products could be found after 7 or 21 days of aging at 37 °C. After 70 days at 37 °C, however, short oligomers at *m/z* 155 and *m/z* 269 were detected. The peak assignments of the molecular ions detected in the LDI-MS spectra are represented in Table 3. The main degradation products appeared as sodium adducts in the LDI-MS spectra. These degradation products are in accordance with a previous study, where the hydrolytic degradation of melt spun and in situ crosslinked PCL fibers was followed [17]. An increase in the number of linear oligomers detected was generally observed with increasing hydrolysis time, temperature and degree of crosslinking. This agrees with previous studies where degree of crosslinking was shown to increase the hydrolysis rate and formation of oligomers due to the lower degree of crystallinity [13,31].

### 3.3. Changes in Functional Groups

**Figure 3:**
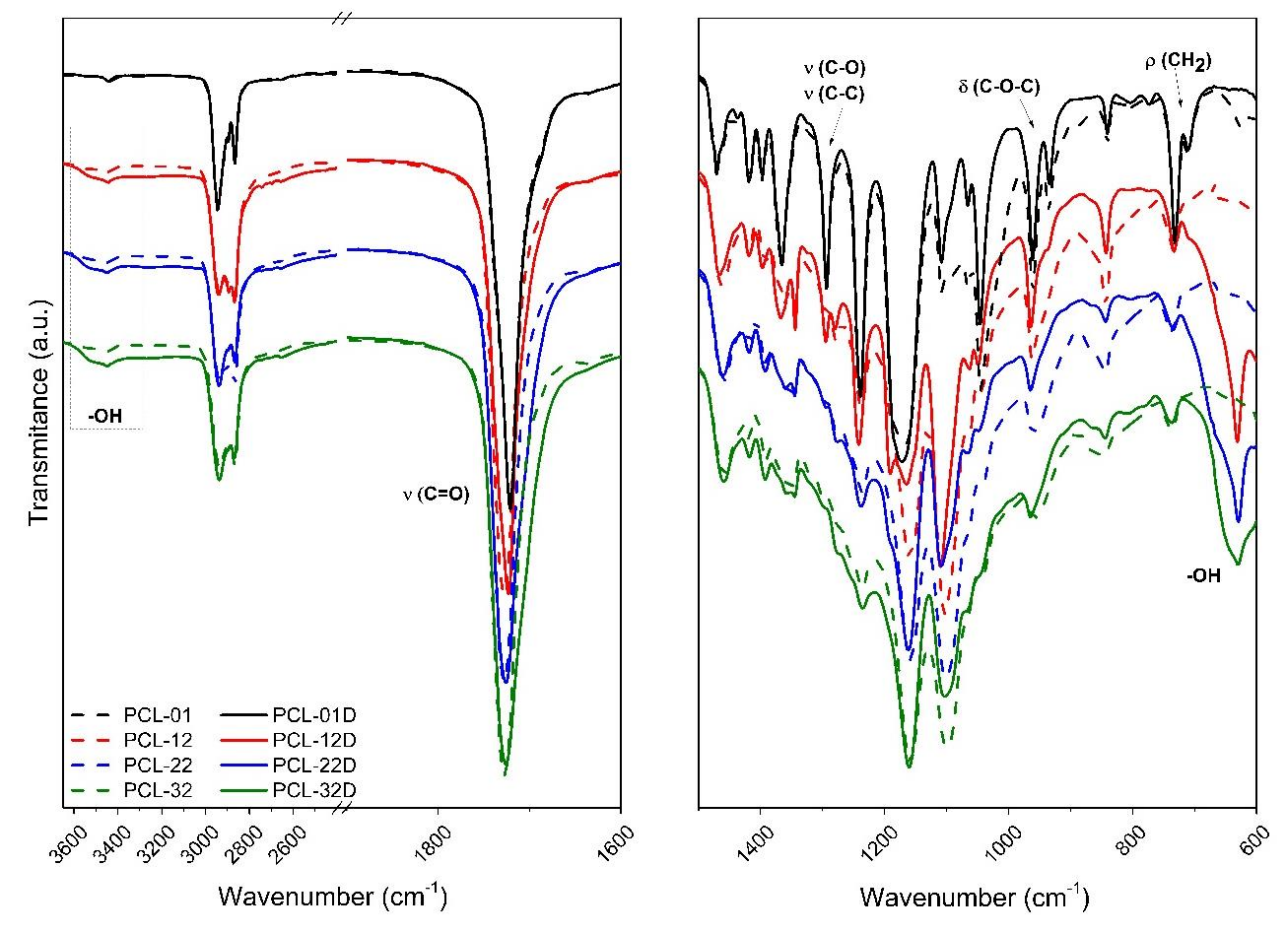
Fourier transform infrared spectroscopy (FTIR) spectra of PCL-12, PCL-22, PCL-32 and the non-crosslinked PCL-01 before and after hydrolytic degradation (denoted with a D) for 70 days at 60 °C.

FTIR analysis of the scaffolds showed that the volume fraction of internal phase did not cause significant differences in the FTIR spectra of the scaffolds, see Figure 3. The crosslinking density governed by the amount of added BCY however caused some small changes in the fingerprint region 1500–600 cm^−1^ in the FTIR spectra. This region is sensitive to the crystallinity degree of the material and to methylene and ester groups vibration in the polymer chains [32,33,34,35]. PCL-12, PCL-22, PCL-32 scaffolds, with medium volume fraction of internal phase and the non-crosslinked PCL-01 scaffold were therefore selected as representative materials to illustrate the differences caused by different crosslinking densities. The FTIR-ATR spectrum of semi-crystalline PCL shows contributions from both crystalline and amorphous phases. The region 2950–2860 cm^−1^ corresponds to the CH_2_ group asymmetric and symmetric axial deformations (*ν*(C-H)). The most evident peak is associated with C=O bond stretching at 1730–1720 cm^−1^. The band at 1293 cm^−1^ can be assigned to *ν*(C–O) and *ν*(C–C) of the polymer main chain in its crystalline form [32]. The low degree of crystallinity is probably the reason for the non-detection of 1293 cm^−1^ band in the most crosslinked scaffolds spectra i.e., PCL-22 and PCL-32 [33]. The broad but weak band at 960 cm^−1^ of crosslinked PCL samples splits into two others when the crystallinity increases: one intense and narrow peak at 960 cm^−1^ and one at 940 cm^−1^ (assigned to ester bond, *δ*(C–O–C)). Likewise, the peak at 740–730 cm^−1^ (attributed to *ρ*(CH_2_)) is also sensitive to the polymer crystallinity, dividing into two other peaks (732 and 711 cm^−1^) in the PCL-01 scaffold [33,36,37].

Significant changes in the FTIR spectra after degradation were only observed for samples hydrolyzed for 70 days at 60 °C, which are shown in Figure 3. The main differences were the clear increase in the intensity of the hydroxyl absorption bands (O–H stretching and O–H out-of-plane bending, at 3600–3300 cm^−1^ and the strong band at 710–600 cm^−1^, respectively) and the broadening of C=O stretching absorption band (1730–1720 cm^−1^). This indicates formation of hydroxyl end-groups as a result of cleavage of the macromolecular chains caused by hydrolytic degradation. After hydrolysis, PCL-12D showed significant changes in the peaks related to the scaffold crystallinity. The peak assigned to *ν*(C–O) + *ν*(C–C) of crystalline form appeared at 1295 cm^−1^. The peaks at 964 cm^−1^ and 734 cm^−1^ were split into two other peaks each, 964 and 936 cm^−1^, and 734 and 710 cm^−1^, respectively. This indicates increasing crystallinity, which is commonly observed during hydrolysis.

### 3.4. Morphological Changes Caused by Hydrolysis

**Figure 4:**
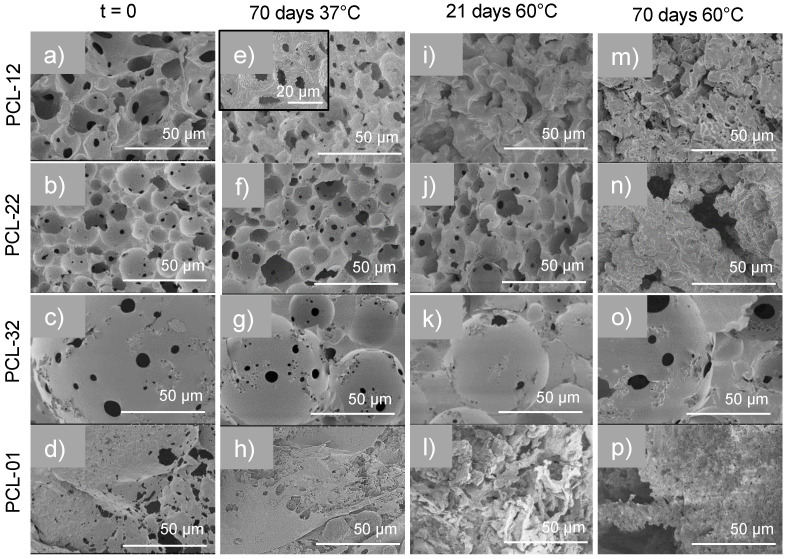
SEM micrographs of the surface of the degraded samples before degradation (t = 0) (**a**–**d**), after 70 days at 37 °C (**e**–**h**), 21 days at 60 °C (**i**–**l**) and 70 days at 60 °C (**m**–**p**).

The morphological analysis by SEM revealed high porosity and interconnectedness for the PCL-based polyHIPEs, see Figure 4a–c. The non-crosslinked PCL on the other hand lacked the interconnectedness and was significantly less porous than the crosslinked samples (Figure 4d). Previous studies have shown that the degradation rate is decreased by increasing the porosity and pore size [13,30,38,39], although this effect could be counteracted by decreasing degree of crystallinity in the case of crosslinked samples. When the volume fraction of the internal phase increased (from 0.74 to 0.85) the void diameters became larger and the overall porosity increased [29]. The effect of crosslinking density on the morphologies was smaller compared to *ϕ*_2_.

The hydrolytic degradation had an apparent effect on the morphologies of the scaffolds (Figure 4a–p). After 7 days at 60 °C all the crosslinked samples retained (more or less) their original morphology, while the PCL-01 already lost its structure. After 21 days at 60 °C all the samples had started to change their appearance as the voids and pores had started to disappear. The original morphology and pore structure had been totally lost for the crosslinked samples with *D_x_* 10% and non-crosslinked PCL-01 (Figure 4i,l). The samples with higher degree of crosslinking, i.e., samples with *D_x_* 20% and 30%, still partly retained their original morphology (Figure 4j,k). Changes were also visible after 70 days at 37 °C for the samples with lower degree of crosslinking (PCL-12) (Figure 4e). The morphologies of the samples with higher degree of crosslinking remain unaffected during aging at 37 °C (Figure 4f,g). However, at 60 °C only the scaffolds with the highest degree of crosslinking *D_x_* 30% (PCL-32) displayed an intact morphology after 70 days (Figure 4o). The non-crosslinked PCL-01 scaffolds displayed significant morphological changes. After 70 days at 37 °C and after 21 days at 60 °C, the pores had been eliminated (Figure 4h,l,p).

## 4. Discussion

The degradation of PCL and its modifications under different aging conditions has been the topic of many investigations [40]. The purely chemical hydrolysis of PCL proceeds slowly due to the relatively long hydrophobic aliphatic segment between the ester groups in the PCL main chain and the high degree of crystallinity [11]. Biodegradation under favorable conditions in e.g., soil or compost can proceed significantly faster [11,41,42,43]. However, the discussion here will concentrate on chemical hydrolysis. The cleavage of ester bonds in the PCL chain through reaction with water, gradually reduces the molecular weight and produces molecules with carboxyl acid and hydroxyl end-groups leading finally to formation of water-soluble degradation products [10]. Although following molecular weight changes was not possible in the present study due to the crosslinked nature of the scaffolds, this process was still clearly observed as the FTIR analysis showed the formation of hydroxyl end-groups and the LDI-MS measurements revealed generation of short oligomers and the monomer, 6-hydroxyhexanoic acid (*m*/*z* 155).

**Figure 5:**
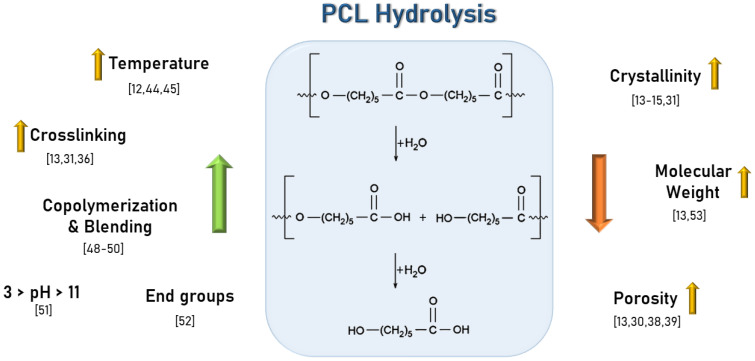
Variables reported for tuning the degradation rate of PCL.

There are several ways to tune the degradation rate by tailoring the chemical configuration, molecular weight and macroscopic design, see Figure 5 for a graphical illustration of some of the established governing factors for hydrolytic degradation process. Temperature is known to have significant effect on the rate of hydrolytic degradation [12,44,45]. Here two different aging temperatures, 37 °C and 60 °C, were utilized. The latter to accelerate the inherently slow hydrolysis rate of PCL. This was deemed necessary as previous studies report a slow hydrolysis rate for crosslinked PCL networks with 10% theoretical crosslinking density with limited changes still after 147 days of hydrolysis at 37 °C [46]. Higher molecular weight generally decreases the hydrolytic degradation rate leading to longer time required for release of water-soluble degradation products. Crosslinked materials, as in the present study, theoretically have an infinitely high molecular weight which counteracts hydrolytic degradation. However, this negative effect is more than compensated by the generally low crystallinity of crosslinked materials, which has been shown to greatly facilitate the hydrolysis rate. This is explained by the easier water penetration due to lower density with randomly oriented amorphous chains making ester groups accessible to water molecules [13,14,15,31]. The effect of crystallinity was also clearly observed here as the scaffolds with higher crosslinking densities and lower crystallinity released more water-soluble products. This was further facilitated by increasing hydrolysis time and temperature. Although higher crosslinking density increased hydrolysis rate, it also helped to keep the morphology of the porous scaffolds longer. Studies using crosslinking to tailor degradation have been reported before. Aminlashgari et al. showed that porous BCY-crosslinked PCL films did not exhibit any significant morphological changes during 3 weeks of hydrolysis at 37 °C or 60 °C, while after 7 weeks minor changes started appearing [17]. On the other hand, PCL fibers that were in-situ crosslinked during an electrospinning process only kept their morphology during aging at 37 °C, while they rapidly lost their shape during hydrolysis at 60 °C. This low stability was attributed to incomplete crosslinking during electrospinning. This was further confirmed by rapid migration of crosslinking agent BCY from the electrospun fibers. Such migration was not observed for the porous PCL scaffolds that were more completely crosslinked during ring-opening polymerization. In accordance, during the present study no crosslinking agents were identified among the water-soluble products and the porous scaffolds with a higher degree of crosslinking kept their morphology longer even at the relatively high hydrolysis temperature of 60 °C. This indicates effective crosslinking reactions during the HIPE process. Another study by Castilla-Cortázar reports superficial surface erosion of PCL scaffolds after 26 weeks of degradation similar to what was observed for the scaffolds with low and medium degree of crosslinking in this study. After 59 weeks authors observed a fibrous structure which was suggested to indicate a certain amount of degradation in bulk as a consequence of the penetration of the degradation media inside the sample [47]. The effect of porosity was addressed in a study reported by Höglund et al. where it was found through a comparison between the hydrolytic degradation of linear homogenous discs, porous structures and crosslinked networks that porous structures together with high molecular weight have shown to yield in a higher resistance towards hydrolysis. This is believed to be due to larger surface areas and thinner pore walls that enables the diffusion of acidic degradation products from the material to the aging solution, consequently suppressing the auto-catalyzed hydrolysis process [13]. However, the porosities of the scaffolds in this study did not differ significantly making it difficult to study its effect in this case and whether it would counteract the observed morphological stability for the highest crosslink density scaffolds. In addition to changes in crystallinity [13,14,15,31], crosslinking density [13,31,36] and macroscopic size and shape [13,30,38,39] discussed above, the degradation rate can be further tailored by e.g., copolymerization or blending [48,49,50], changing degradation media [51], end-group modification [52] or by adjusting molecular weight [53].

## 5. Conclusions

The influence of crosslinking density and volume fraction of dispersed phase on hydrolytic degradation process of PCL scaffolds prepared by HIPE-ROP was evaluated. The effect of crosslinking was two-fold. On one hand crosslinking resulted in lower crystallinity, which increased the release of water-soluble hydrolysis products as indicated by the increasing *m/z* signal intensities in the LDI-MS spectra. At the same time, the porous structure and morphology was retained longer, the more crosslinked the material was. The volume fraction of dispersed phase did not influence the hydrolytic degradation process as significantly. Degradation products in the form of linear oligomers ranging from dimer to hexamer were detected by LDI-MS. The amounts of water-soluble products increased with increasing hydrolysis time, temperature and crosslinking density.

## Figures and Tables

**Table 1:** High internal phase emulsion (HIPE) formulations for the synthesis of crosslinked poly(ε-caprolactone) (PCL) scaffolds.

Sample ID	CL (g)	*D_x_* (%) *	BCY (g)	Sn(Oct)_2_ (g)	*ϕ*_2_
PCL-11	1.1	10	0.12	0.041	0.74
PCL-12	1.1	10	0.12	0.041	0.80
PCL-13	1.1	10	0.12	0.041	0.85
PCL-21	1.1	20	0.27	0.045	0.74
PCL-22	1.1	20	0.27	0.045	0.80
PCL-23	1.1	20	0.27	0.045	0.85
PCL-31	1.1	30	0.47	0.049	0.74
PCL-32	1.1	30	0.47	0.049	0.80
PCL-33	1.1	30	0.47	0.049	0.85

* Calculated using equation *D_x_* = [2*a*/(2*a* + *b*)] × 100, where a and b are mole fractions of bis(ε-caprolactone-4-yl) (BCY) and ε-caprolactone (CL), respectively.

**Table 2:** Thermal properties, density and porosity values of crosslinked PCL scaffolds.

Sample ID	*T*_m_, (°C) *	Δ*H_f_*, (J/g) *	Crystallinity (%) *	Density (g/cm^3^)	Porosity (%)
PCL-01	55.8	51.8	38.1	-	-
PCL-11	35.1	21.5	15.8	0.45 ± 0.01	60.5 ± 1.2
PCL-12	38.1	17.2	12.6	0.33 ± 0.03	71.4 ± 3.7
PCL-13	33.6	7.7	5.7	0.29 ± 0.02	74.7± 2.5
PCL-21	34.2	19.3	14.2	0.46 ± 0.04	60.1 ± 5.0
PCL-22	33.7	14.9	11.0	0.42 ± 0.02	63.3 ± 2.5
PCL-23	34.2	13.3	9.8	0.30 ± 0.02	73.5 ± 2.5
PCL-31	33.6	17.9	13.2	0.66 ± 0.01	42.2 ± 1.2
PCL-32	32.7	16.2	11.9	0.46 ± 0.03	60.0 ± 3.7
PCL-33	31.6	13.6	10.0	0.37 ± 0.02	67.6 ± 2.3

* Melting temperature (***T*_m_**), heat of fusion (**Δ*H_f_***) and degree of crystallinity determined from the second heating cycle of DSC.

**Table 3:** Peaks assignments with *m/z* of linear water-soluble degradation products observed by laser desorption ionization-mass spectrometry (LDI-MS).

	*m*/*z*	Structure
**Linear products:**	155	H–[CL]_1_–OH + Na^+^
	269	H–[CL]_2_–OH + Na^+^
	360	H–[CL]_3_–OH
	383	H–[CL]_3_–OH + Na^+^
	497	H–[CL]_4_–OH + Na^+^
	611	H–[CL]_5_–OH + Na^+^
	725	H–[CL]_6_–OH + Na^+^
